# Protocol for an aged cohort study to create a single indicator that expresses the trajectory of intrinsic capacity over the years and its relation to functional abilities

**DOI:** 10.3389/fragi.2025.1459284

**Published:** 2025-10-15

**Authors:** E. J. Simões, M. Perracini, M. A. F. Mello, F. M. Cohrs, A. P. F. M. Neumann, M. Demarzo, L. R. Ramos

**Affiliations:** ^1^ School of Medicine, University of Missouri, Columbia, MO, United States; ^2^ Universidade Cidade de São Paulo, São Paulo, Sao Paulo, Brazil; ^3^ Paulista School of Medicine, Federal University of São Paulo, São Paulo, São Paulo, Brazil

**Keywords:** aged, intrinsic capacity, activities of daily living, health status indicators, longitudinal studies, functional status, frailty

## Abstract

Population aging will be on the public health agenda in the coming decades. By 2050, 16% of the world’s population will be aged 65 and above, mostly living in middle- and low-income countries. In Brazil, the aged population will triple by 2050, from less than 20 million to approximately 65 million, making it the sixth largest aged population in the world. Population aging is associated with an increase in the prevalence of chronic non-communicable diseases, which in turn promotes a functional decline in people who age. This often leads to limitations in daily life and dependence, with clear implications for the quality of life and health costs. In 2015, the World Health Organization proposed the concept of intrinsic capacity (IC) as a multidimensional health indicator that encompasses the essential physical and mental capabilities for people to perform what they need and like in daily life, regardless of the chronological age. In practice, IC was operationalized in five health domains, namely, cognitive, psychological, sensory, locomotor, and vitality. These domains, which are evaluated together and over time, offer the function parameters necessary to understand different, person-centered aging trajectories. Prevention of age-associated functional decline has not been studied well. Literature lacks studies that indicate the expected values for different IC trajectories related to aging, with or without disability. Few studies have analyzed IC as a risk factor for compromising the functional ability (FA) of the elderly, which is measured by the degree of dependence in activities of daily living, the risk of falls, and early mortality, while controlling for all known risk factors for functional decline. The cohort study proposed here, called “Longevity with Functionality (LONGFUN),” addresses the growing importance of evaluating the indicators of IC in a prospective way, creating a single indicator, and relating it to the FA of the aged population.

## Introduction

Population aging will be on the public health agenda in the coming decades. By 2050, 16% of the world’s population will be aged 65 and above, mostly living in middle- and low-income countries. In Latin America and the Caribbean, estimates indicate that this age group will represent 19% of the entire population (World Health Organization, 2015). In Brazil, the population aged 60 and over will triple by 2050, increasing from less than 20 million to approximately 65 million (15 million aged 80 or over), making it the sixth largest elderly population in the world (Ramos et al., 1987; Fapesp, 2016).

While population aging represents an improvement in the population’s social and health indicators, there are countless challenges accompanying this demographic transition. For example, longevity lived with good functionality, one of the pillars of quality of life (QoL) among the elderly, will be a privilege for some, but many will experience limitations in their daily lives in the final third stage of life (Rosa et al., 2003).

Population aging is associated with an increase in the prevalence of chronic non-communicable diseases (NCDs), which in turn promotes functional decline in people who age; this often leads to limitations and dependence in daily life, with clear implications for the QoL and healthcare costs (Calderón-Larrañaga et al., 2019; Costa Filho et al., 2018; Miranda et al., 2016; Ramos, 2003). The cohort study proposed here, called “Longevity with Functionality (LONGFUN),” addresses the growing importance of evaluating the rate of progression of functional limitations with age and factors associated with the loss of the ability of individuals to live with independence and autonomy.

### Prevention of functional losses

Prevention of age-associated functional decline is still not well-studied. Some existing studies show that it is possible to increase the physical activity in sedentary elderly people (Novais et al., 2019) and the cognitive ability of elderly people with mild cognitive impairment through digital inclusion workshops (Irazoki et al., 2020; Vicentin et al., 2018). We can also improve the mental state of older adults with mindfulness in late-life depression (Mapurunga et al., 2020; Mathur et al., 2016) and aid in hearing and vision with increasingly accessible prostheses (Lin et al., 2013; Whitson and Lin, 2014). In addition, physical independence can be improved with assistive technology, which range from the simplest (cane) to the most sophisticated (robots) (Haufe et al., 2019; Lichtenberg, 2024).

### Intrinsic capacity

In 2017, the World Health Organization (World Health Organization, 2017) proposed the concept of intrinsic capacity (IC). This is a multidimensional health indicator that encompasses the essential physical and mental capabilities for people to perform what they like, regardless of their chronological age. This approach modifies the negative stereotypes linked to old age by emphasizing the positive attributes of health and the physiological reserve that individuals can develop and maintain throughout life.

In practice, IC has been operationalized in five domains: vitality, mobility, sensory, cognitive, and psychological. These domains, which are evaluated together and over time, offer the functional parameters necessary to understand different, people-centered aging trajectories during the Decade of Healthy Aging (2020–2030) (Dixon, 2021; Rudnicka et al., 2020).

## Rationale

Research shows that the prevalence of age-related functional disability in Brazil is large and still growing (Giacomin et al., 2018; Lima-Costa et al., 2022), but we do not know if this phenomenon is occurring in terms of IC. Recent research shows that IC is a valid measure for healthy aging in a country such as Brazil (Aliberti et al., 2022). A Latin American study showed that the reduction of IC in old age, including one or more of the factors in IC’s five domains, has a negative impact on the functional ability (FA) in the elderly (Prince et al., 2021). These associations must be reassessed with longitudinal studies in new environmental contexts such as the elderly population in Brazil (Martin and Ortuño, 2019).

Literature lacks studies with IC as the outcome variable, which is needed to indicate the normality values for different IC trajectories related to aging, with or without disability. Few studies have analyzed IC as a risk factor for compromising the FA of the elderly, measured by the degree of dependence in the performance of day-to-day activities, the risk of falls, and early mortality, while controlling for all possible known risks for functional losses—clinical, biological, genetic, and social (Prince et al., 2021). There are studies that point out that IC can predict the decline in activities of daily living (ADL) and instrumental activities of daily living (IADL) even after adjusting for sociodemographic factors and chronic diseases (Lu et al., 2021; Prince et al., 2021; Fong, 2019; Jonkman et al., 2018; Pan et al., 2021).

The key medical literature gaps that this proposed research aims to fill is how a set of comprehensive factors such as comorbidity, medications, biomarkers, pain, nutrition, strength, balance, gate, physical environment, hearing, vision, cognitive ability, motivation, and behavior, along with contextual factors such as demographic and socioeconomic factors, especially in Brazil, may work synergistically to impact ADL/IADL, falls, hospitalizations, and death as people age. The IC measure and its domains capture the complexity of the 14 factors’ interactions, allowing for the evaluation of the effect of not only each factor but also their joint effect.

In addition, there is a pressing need in healthcare services for studies that assess the impact of effective health promotion strategies—cognitive stimulation, promotion of physical activity, and nutritional education—in preventing IC loss and maintaining FA in elderly people in Brazil. For this purpose, longitudinal observational studies are needed to initially assess these scientific assumptions and justify and guide future studies of controlled interventions.

This paper describes a proposal to 1) constitute a population cohort of elderly people living in a middle-class neighborhood in a large urban center in Brazil and 2) create a single indicator that expresses the trajectory of IC over the years. For this purpose, an assessment of the IC, the FA, and the social capital of the members at baseline and after 3 years will be carried out, establishing an average rate of IC decline, while controlling for the risk and protective factors—genetic, biological, clinical, behavioral, and environmental. The results will be vital for designing future interventional studies that prevent or mitigate functional ability losses associated with age. We intend, through the results from this cohort, to create an inclusive ecosystem of translational innovation in longevity integrated into a national/global innovation system in a continuous process of development and partnerships.

### Objectives

The cohort study proposed here addresses the determinants of IC and its relationships with FAs that allow longevity with good health and independence in everyday life. Considering that the literature on cohort studies that indicate IC values for the different aging trajectories is limited, we intend to build an IC index composed of the different domains, which can be validated as a risk indicator for classic outcomes such as hospitalization, falls, death, and severe dependence. We also intend to use the data to design clinical trials and nested case–control studies, with sample power, to evaluate measures aimed at preventing or mitigating functional ability losses over time.

To that end, we aim to

1. Create a population cohort of people aged 60+ (LONGFUN) living in a middle-class neighborhood in a large urban center in Brazil.
2. Create a composite IC index considering the five domains: cognitive, psychological, sensory, locomotor, and vitality.
3. Carry out an epidemiological segmentation considering the index composed of IC and the sociodemographic, behavioral, and clinical factors.
4. Identify the genetic and immunological biomarkers associated with each segment in a representative subsample of the cohort to be evaluated by a geriatric and multidisciplinary team that will define the needs of care for each segment.
5. Analyze the trajectory of the IC composite index for the total cohort and for each segment created, seeking its predictive power as an indicator of risk for the main outcomes: hospitalization, disability, falls, and death.
6. Monitor IC changes over time and the impact of the changes on baseline predictors.
7. Propose strategies for the development of an integrated system for people-centered healthcare based on the identified needs for care.

## Methods

### Study design

The proposed study is a 3-year prospective cohort study of a population of older adults aged 60 years—the onset age designated by the World Health Organization (2015)—or older in the city of São Paulo, Brazil. The result will be a set of biological, social, clinical, and functional data that will allow the study of the progression of IC over time among this population and its associations with ADL, IADL, falls, hospitalization, and death.

### Study area and population

This cohort study is being proposed in the coverage population of the Basic Health Unit (UBS) Vila Mariana in the Southeast Health Coordination, an area adjacent to the area of influence of Hospital São Paulo in the Districts of Health and Vila Mariana in the city of São Paulo, where the previous cohorts of the EPIDOSO Project were selected (Ramos et al., 1998; Ramos et al., 2013). The study area has 156 census sectors (SC) and 39,104 households.

Based on the 2010 census of 100,000 inhabitants, approximately 15,000 seniors (aged 60+) residing in this area will be eligible for this study. The eligible and potential participants of the cohort represent an urban population with a good educational and socioeconomic level.

### Eligibility

All adults aged 60 and older living in the study area who will be contacted by the study team can participate in the study. Older adults residing in long-stay institutions for the elderly and those who are bedridden will be excluded from our study as the focus of this research is the follow-up of IC until the development of one or more outcomes: severe dependence, fall, hospitalization, or death as a function of IC. It will also be important to ensure through an initial in-house geriatric assessment that participants at baseline do not have significant loss of independence in the activities of daily living (ADL and IADL scores >0) (Katz and Akpom, 1976; Lawton and Brody, 1969) and cognitive ability (Clinical Dementia Rating (CDR) > zero) (Morris, 1994).

### Study sample size and power calculation

**FIGURE 1:**
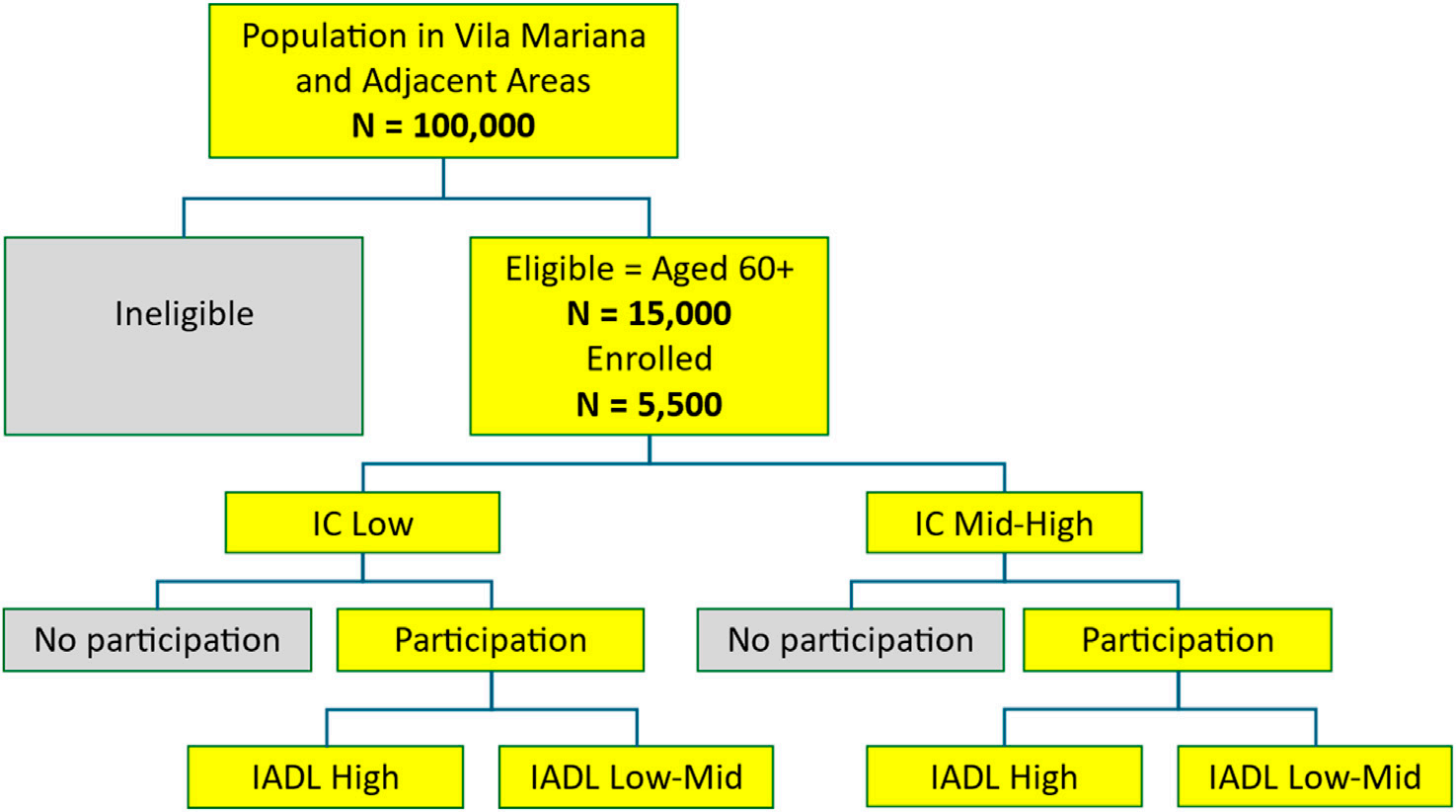
Proposed study design.

A census of the study area will randomly select 50 census sectors (CSs). All adults aged 60 years or older residing in these sectors will be invited to enroll in the cohort through a door-to-door visit (≈6,000 seniors ≥60 years initially listed). All those who will have been selected and would agree to participate and enroll will be evaluated with geriatric and gerontological assessment, including a battery of clinical exams. Based on the experience of previous cohorts, a refusal rate of ≈15% is expected from the older adults contacted, for an initial sample of approximately 5,500 participants (Figure 1).

In a period of 6 months, we expect that a team of trained interviewers will contact all potential cohort participants. Based on information from a recent study on the association of IC with the outcome incidence rates of functional dependence, falls, and death (Prince et al., 2021), we developed sample size estimates for comparing two proportions that represent the rates of outcomes in the groups that are exposed (low IC) and unexposed (medium or high IC) (see Tables 1, 2). Calculations considered various effect sizes (relative risk) and incidence or mortality rates. In estimating the sample size needed for this cohort study, this study considered the incidence of the expected outcome in the unexposed cohort (high IC), the risk assumed in the exposed group relative to the risk of the unexposed group (RR), a specific level of confidence (1-α = 0.95; where α = type I error rate), and a specific value of the desired sample power (1-β = 0.80; where β = type II error rate) for the detection of significant differences between the two cohorts. The outcome incidence rate in the cohort was used as a proxy for the expected outcome incidence rate in the unexposed group (high IC). The summary tables (Tables 1, 2 below) provide sample sizes for extremes of the assumed incidence and mortality rate values and the respective relative risks based on the confidence intervals of these measures. A total sample size of approximately 5,500 individuals (assuming 2,750 per group) will be sufficiently robust to maintain the desired power under most diverse scenarios and varying parameters, even if the dropout rate reaches approximately equal to 15% in the two cohorts. The exception will be with very small estimates of the relative risk of death (RR ≤ 1.3).

**TABLE 1:** Sample sizes for specific values of functional loss (FL), incidence, and relative risk for intrinsic capacity (IC) and FL.

	Average risk ratio	Low risk ratio	High risk ratio
Risk ratios (RR)	1.91	1.7	3.0
Incidence rate among the unexposed group	0.0495	0.04925	0.04925
Confidence level (1-α)	0.95	0.95	0.95
Power (1-β)	0.8	0.8	0.8
Study type	Cohort	Cohort	Cohort
Sample per group (n_i_)	514	816	138
Total sample (n_1_+n_2_)	1,028	1,632	276

**TABLE 2:** Sample sizes for specific values of total mortality (TM) and relative risk for IC and TM.

	Average risk ratio	Low risk ratio	High risk ratio
Risk ratios (RR)	1.66	1.3	2.0
Mortality rate among unexposed	0.04826	0.04826	0.04826
Confidence level (1-α)	0.95	0.95	0.95
Power (1-β)	0.8	0.8	0.8
Study type	Cohort	Cohort	Cohort
Sample per group (n_i_)	926	3,922	432
Total sample (n_1_+n_2_)	1,852	7,844	864

### Instruments and measures

**FIGURE 2:**
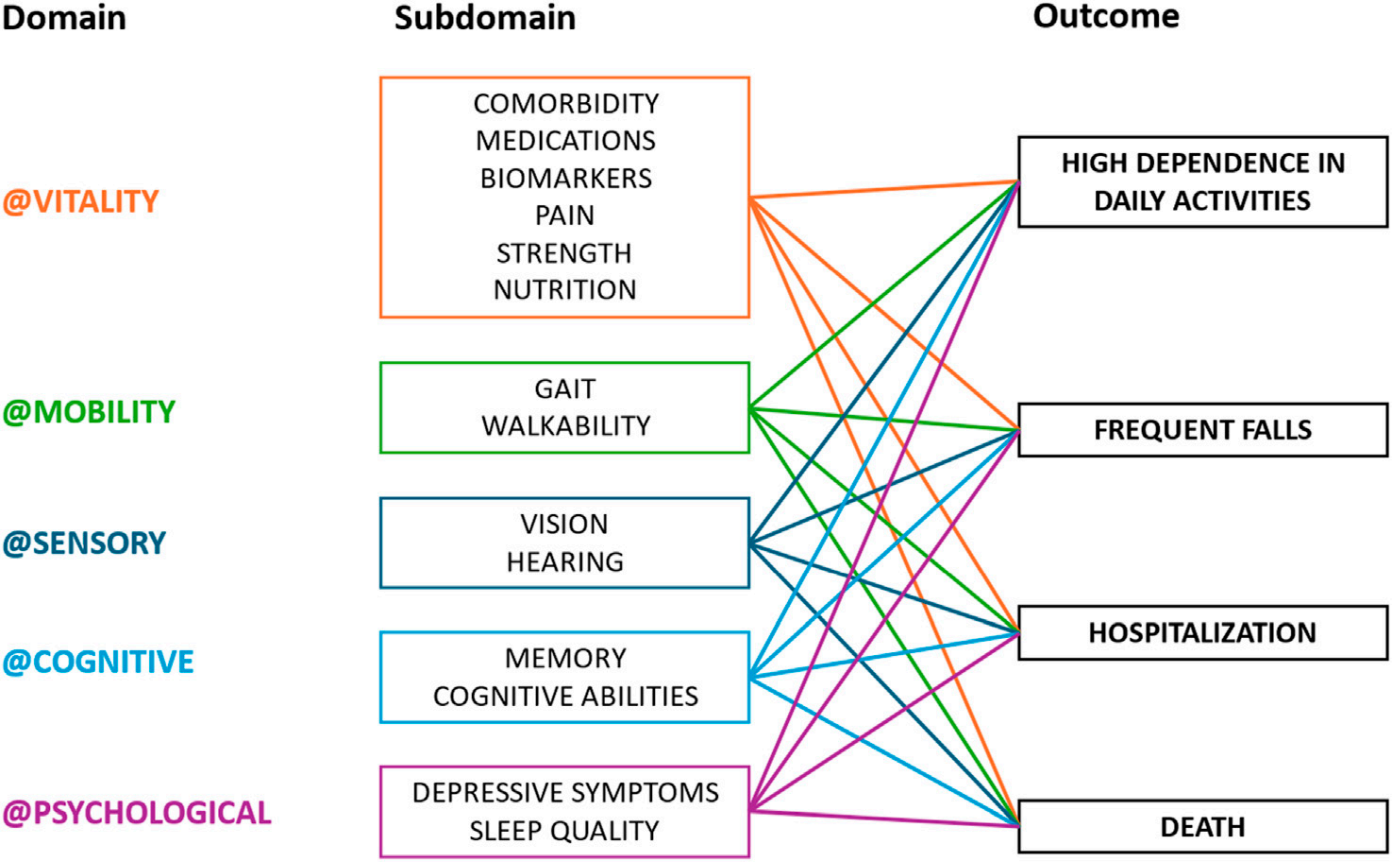
Intrinsic capacity: its domains, subdomains, and related health outcomes.

To assess IC and its relationship with FA in older adults, a range of clinical and behavioral factors will be investigated through comprehensive geriatric and gerontological interviews and laboratory tests (Figure 2). In summary, the main variables of this research are as follows:

## Dependent variables

• Need for help in ADL (Katz and Akpom, 1976) and IADL (Lawton and Brody, 1969).
• Falls in the last year.
• Hospitalizations in the last year.
• Deaths.

Explanatory variables: IC domains and subdomain measures.

The IC subdomain measures, their levels, cut off points for a binary classification, and references are presented in Table 3.

**TABLE 3:** Intrinsic capacity domains and subdomains measures with classification cut-offs.

Domain	Subdomain	Classification (binary: 0 or 1)	References
Vitality	Number of chronic morbidities	<2 = 0 | ≥ 2 = 1	Charlson et al. (2022) and Charlson et al. (1987); Kim et al. (2021)
	Number of medications taken daily	0–4 = 0 | 5+ = 1	Golchin et al. (2015)
	Handgrip strength	Men:<=36.2 kg = 0 | > 36.2 kg = 1; Women:<=23.0 kg = 0 | > 23.0 kg = 1	Amaral et al. (2019), Alqahtani et al. (2019)
	Body mass index	≤ 25 = 0 | > 25 = 1	Kiskaç (2022), Krzyminska-Siemaszko et al. (2014)
	Biomarkers of metabolism and inflammation	NL = 0 | high = 1	Liu et al. (2017), Bautmans et al. (2022), Bohannon (2019)
	Pain numerical rating scale (NRS)	≤ 6 = 0 | > 6 = 1	Devin and McGirt (2015), Atisook et al. (2021)
Mobility	Tandem stance	Tandem stance for>=10 s = 0 | tandem stance for <10 sec= 1	Joo et al. (2021)
	Gait (timed up and go)	<6 min = 0 | ≥ 6 = 1	Herman et al. (2011); Alexandre et al. (2012)
Sensory	Community screening for visual impairment	Good = 0 | impaired = 1	Clarke et al. (2018)
	Hearing screening in older adults	Good = 0 | impaired = 1	https://hign.org/consultgeri/try-this-series/hearing
Cognitive	Memory (MOCA)	≥ 25 = 0 | < 25 = 1	Nasreddinne et al. (2005)
	Cognitive abilities (CDR)	0 = 0 | ≥ 0,5 = 1	Morris (1994)
Psychological	Depressive symptoms (GDS)	<5 = 0 | ≥ 5 = 1	Almeida and Almeida (1999), Yesavage et al. (1983)
	Sleep quality	good = 0 | bad = 1	Smyth (2013)

### Explanatory variables: IC composite index (ICI)

An IC composite score or index (IC index or ICI) will first be created, as suggested by Gutiérrez-Robledo et al. (2019). This score aims to express a summary measure of impairments for an older adult. Initially, each continuous or ordinal subdomain measure will be dichotomized based on acceptable cut-off points to create a binary variable of 0 or 1 (see Table 3 for cutoff points across all subdomain measures). Within a domain, the values of these recoded subdomain measures will be summed up to create a “subdomain score.” The subdomain scores will range from 0 to 2 for the four subdomains with only two measures and from 0 to 6 for the subdomain with six measures (i.e., vitality). Then, each domain of the five IC components will be subdivided into three categories based on the sum of the scores of its subdomains, with the following numerical score: no impairment = 0, mild impairment = 1, and severe impairment = 2. For example, the four domains with only two subdomain measures (e.g., cognitive with subdomain measures memory and cognitive) will be coded 0 if their subdomain score (sum of their subdomain measure) is 0, 1 if the subdomain score value is 1, and 2 if the subdomain score value is 2. The domain with six subdomain measures, vitality (subdomains comorbidity, medication, biomarkers, pain, strength, and nutrition), will be coded 0 if its subdomain score is 0, 1 if the subdomain score is 2–4, and 2 if the subdomain score is 5–6. Scores for each of the five domains will be summed up to produce a composite IC index ranging from 0 (best possible ICI) to 14 (worst possible ICI).

Subsequently, this baseline ICI will be classified in four ways. First, using the terciles of ICI (TIC), ICI values less than or equal to the first tercile (Q1) will be classified as low TIC; ICI values between Q1 and the third tercile (Q3), including Q3, will be classified as medium TIC; and ICI values above Q3 will be classified as high TIC. This analysis model is explicitly shown in Figure 1.

Second, the change in the ICI value during the follow-up time in the cohort will be measured and referred to as “ICI delta” (∆IC). The ∆IC is calculated as the difference between the ICI measured in the last follow-up wave minus the ICI measure at the baseline (variable with values between 0 and 10). Third, we will consider the presence of a decline in IC (DIC) if one or more declines in an IC domain are found from the baseline measure. As an example of IC domain decline in the cognitive dimension, a Montreal Cognitive Assessment (MoCA) score of 22 at follow-up versus 27.0 at baseline is considered a decline and probable “dementia” (Nasreddine et al., 2005). The opposite will be classified as no decline.

Finally, a weighted measure of IC (WIC) will be derived from the multivariate regression result of the association between IC subdomains and IADL loss, which will be adjusted for individual sociodemographic factors (see the Analysis section below for detailed description of WIC).

### Other variables of interest

• Sociodemographic (age, sex, and race)
• Socioeconomic (work and income)
• Social capital (number of family and friends)
• Quality of life (QoL indicator extracted from the World Health Organization Disability Assessment Schedule 2.0 (WHODAS)) (Ferrer et al., 2019; Üstün, 2010)
• Physical activity (leisure-time physical activity (LTPA) indicator extracted from the International Physical Activity Questionnaire (IPAQ)) (Craig et al., 2003).

## Research questions and hypotheses to test

This proposed research will answer three scientific questions:

1. Whether IC will decrease as expected at the 3-year follow-up (ICI at end-time ≤ ICI at baseline, or ∆IC < 0), regardless of the socioecological context, individual sociodemographic, behavioral, and clinical factors, and comorbidities (Charlson et al., 1987). This research will statistically test the hypothesis that ICI will not change over time (i.e., follow up IC = baseline IC, or ∆IC = 0).
2. If DIC (or TIC) at baseline will be a strong predictor of FA, such as ADL (Mlinac and Feng, 2016), IADL (Hokoishi et al., 2001), falls (Kitcharanant et al., 2020), and death, regardless of the socioecological context, individual sociodemographic, behavioral, and clinical factors, and comorbidities (Charlson et al., 1987). That is, whether the proportion of these ADL/IADL losses, falls, and deaths will be greater in the decline group (e.g., DIC present) than in the unexposed group (e.g., DIC absent). Research will statistically test the hypothesis that there is no association between IC and ADL (or IADL) (e.g., relative risk for TIC or DIC and IADL = 1).
3. Whether IC and its five domains are directly associated with a measure of QoL (i.e., World Health Organization Disability Assessment Schedule 2.0 (WHODAS 2.0)) (Prüss-Ustün et al., 2017) and LTPA (i.e., the International Physical Activity Questionnaire (IPAQ)) (Craig et al., 2003), regardless of the socioecological context, individual sociodemographic, behavioral, and clinical factors, and comorbidities (Charlson et al., 1987). Research will statistically test the hypothesis that there is no association between IC and either QoL or LTPA (e.g., relative risk for TIC or DIC and LTPA = 1).

### Data analysis

The analysis will be implemented in SAS Institute Inc (2023). For all analyses at baseline and final follow-up time, descriptive statistics (e.g., frequency, central trend, and variability) and diagnostic plots (e.g., histogram or kernel density estimation and q–q plots) will be implemented on all variables. Data for outliers will be evaluated and tested as needed for statistical model assumptions. Appropriate corrective strategies, such as transformations, the use of robust methods, and/or data reduction, will be used if problems are identified.

These preliminary analyses aim to ensure high-quality data and test the assumptions of the proposed statistical models. We assume that this study (cohort) suffers from an uneven distribution of confounding factors, which may be exacerbated by the bias due to the possible unequal dropout rates between exposure groups. Therefore, adequate adjustment of the characteristics of the study subject is crucial to make an unbiased inference of the associations between the outcomes of interest (addiction, falls, and mortality) and the main exposure factor, IC, and the associations between IC and its five domains (vitality, mobility, sensory, cognitive, and psychological) and the evolution of IC in the follow-up time.

It is also expected that not all values for all variables will be collected, thus generating observations with missing values. If there is a substantial number of unused cases under analysis due to missing values (≥5%), multiple imputations of these values will be used along with a corresponding sensitivity analysis. In the sensitivity analysis, models with the inclusion of imputed missing data will be compared to original models in which missing data are not included. If there is a substantial change in the parameters of interest between these two models (e.g., a nonsignificant coefficient in the original model becomes significant in the model with imputed data), the imputed model will be accepted as the least-biased model in terms of the bias introduced by the missing values of variables.

### Generation of weighted IC score (WIC)

To build the weighted IC score (WIC), first, associations between subdomains of IC domains (sensory, mobility, vitality, cognitive, and psychological) and IADL loss will be evaluated by IADL models in a two-stage logistic regression (Meng et al., 2022). For this purpose, IC subdomains will be dichotomized, such as body mass index (BMI) (overweight–obese v. normal–underweight) and handgrip strength (normal–strong v. weak), both of which are subdomains of the vitality domain (Chen et al., 2020). The first stage of modeling requires bivariate analysis between each IC subdomain and IADLs to identify candidate subdomains to enter the multivariate logistic model (Meng et al., 2022). For this, the variable in the model whose *p-*value for the statistical test of association (chi-square) with IADL is less than or equal to α = 0.25 will enter the multivariate logistic regression model with adjustment for the sociodemographic, comorbidity, behavioral, and clinical factors. An exception will be variables selected *a priori* since they are clinically fundamental, such as BMI from the vitality domain.

In the multivariate logistic regression model, subdomains whose *p-*values are less than or equal to α = 0.05 (statistically significant) will be used to generate WIC as follows. For the regression coefficient in the multivariate model that reaches statistical significance (*p ≤* 0.05), its component will be transformed into a subdomain score by dividing the coefficient by the smallest regression coefficient in the model after rounding the absolute value of the coefficient to the nearest integer value. Regression coefficients that are not statistically significant in the adjusted model will have a value of one (1) point. A summary WIC will be obtained by adding points from all regression components. The subdomain terms will enter the multivariate regression model such that the higher the WIC score, the better the IC.

#### Statistical models to assess whether IC is a predictor of ADLs/IADLs, falls, and death

Two Cox regression models will be evaluated to estimate the incidence rates of outcomes (i.e., functional loss measured by IADLs, falls, and death) associated with IC at baseline (e.g., TIC, DIC, and WIC) (ElHafeez et al., 2021). Cox’s first model has the indicator variable X to denote the association with each of the two groups of IC exposure at baseline (e.g., X1 = 1 for medium and high TIC and X1 = 0 for low TIC), with Y being the adverse incident at 3 years of follow-up (loss of ADLs/IADLs, falls, and death); survival times will be measured by the interval from the first assessment to the adverse incident. For subjects who do not experience an adverse incident, the follow-up time will be the last evaluation of the study.

The model also comprises a collection of covariates V with the potential to be a confounding factor in the association between Y and X (e.g., sociodemographic factors), and W would be the potentially interactive term between X and V (e.g., sex * TIC). The regression coefficient of X describes the effect of TIC on outcome Y, that is, the risk of greater or lesser loss of ADL/IADL, falls, and death for those exposed to medium or high TIC relative to those exposed to low TIC. The W terms will assess whether there is a positive interaction between TIC and one of the V confounders that have been identified to be potentially interacting. The model will extend by substituting TIC for DIC or WIC as the main exposure variable X. Alternatively, we can also model Y (e.g., IADL) as a function of X (either TIC, DIC, or WIC) and the same set of V and W using logistic regression to estimate the odds ratio of the outcomes.

We will evaluate the IC trajectory through changes in IC scores (∆IC or WIC) or the presence of IC decline (DIC) after 3 years, along with their associations with IADL outcomes, falls, and death. For example, the mean/median of ∆IC (or WIC) and the prevalence of DIC will be generated at baseline and at the end of the 3-year follow-up. We will then generate the mean/median or prevalence of IC measure by levels of ADL/IADL, falls, and death. The means/medians of IADLs and the incidence of falls and death after 3 years of follow-up will also be estimated.

In contrast, we should also be able to model Y as ∆IC (ordinal variable with values from 0 to 10) as a function of a main vector X (IADL, falls, or death) and a collection of parameters V and W similar to the previous model, but using a multivariate ordinal regression model with a latent variable specification, where composite probability methods are applied to estimate model parameters (Hirk et al., 2019).

#### Statistical models to assess the question of whether the quality of life (WHODAS 2.0) and leisure-time physical activity (LTPM) are associated with IC

A slope coefficient of IC (Y = 1 if DIC = 1 or more declines, and Y = 0 if DIC = no decline) will be estimated by a logistic regression model as a function of QoL at baseline (WHODAS = 0 (high), WHODAS = 1 (medium), or WHODAS = 2 (low)) adjusted for the influence of other individual factors such as sociodemographic factors (age, sex, education, and health coverage), comorbidities, loss of ADL/IADL, and socioecological factors (e.g., sociability). The set of V and W will be a collection of covariates similar to previous models, repositioning X = 1 for LTPA that reaches the recommended levels and X = 0 for LTPA that does not reach recommended levels and keeping the same collection of V and W. The relationship between LTPA and IC (or DIC), adjusted for other individual socioecological factors, will also be evaluated by logistic regression.

## Discussion

There are several reasons for proposing a prospective cohort for the over-60 aged population of São Paulo, Brazil. First, given the rapid aging and the increase in functional limitation among most of the elderly populations in Brazil and the world, it is necessary to better understand the implications of various measures that compose IC and IC itself for the multiple factors that impact on loss of functionality at old age, including preventive and curative actions (Rosa et al., 2003; Costa Filho et al., 2018).

Second, the measure of aging promoted by WHO—IC—has been well-studied in Europe and North America, but the sole study of IC in Latin America did not include Brazil. Brazil has the largest and fastest growing population that is 65 years of age and older in Latin America and the world, and it is predicted to be the fourth largest in the world by 2050. In addition, the complex mix of racial, ethnic, socioeconomic, and environmental factors among urban older populations in Brazil are not easily found elsewhere and provide a fertile ground for exploring multifactorial causes of changes in IC and functional limitations.

Moreover, cohort studies are the strongest methodology for observational epidemiological designs that allow for ethical investigation of IC and its associated events. In this design, all relevant variables and related factors identified in advance can be measured without error and recorded (Andrade, 2022). The design allows for the investigation of multiple outcomes for selected or rare number of exposures. In addition, the cohort study guarantees the maintenance of a temporal sequence between exposure to risk or protective factors and healthcare outcomes, while allowing for the calculation of incidence rates. In addition, prospective cohort studies allow the inclusion of other studies that take advantage of the already formed cohort, such as nested case–controls, and the identification of cohort subsets to evaluate interventions in a randomized intervention trial.

Our proposed prospective cohort study, aiming to evaluate hypotheses about the effect of IC on ADL, IADL, and WHODAS and their progression over time in an older population of São Paulo, Brazil, will be only the second such study to include a Latin American population.

The proposed study may suffer the expected limitations of cohort studies, namely, selection bias, including loss of follow-up and misclassification of exposure, as well as confounding during analysis. The proposed design is less prone to suffer from the possible misclassification of outcomes such as ADL/IADL, hospitalization, frequent falls, and death since their measurements are uncontroversial, scientifically tested, and acceptable (Wæhrens et al., 2021; Prinja et al., 2012; Kitcharanant et al., 2020; Rothman and Greenland, 1998).

In a previous 5-year follow-up cohort study with the participation of 1,600 members from the same eligible population described in this proposal, attrition led to the loss of 500 cohort members in 2 years and 1,000 members by the end of the study period (Ramos et al., 2001; D’Orsi et al., 2011). In order to minimize the biggest threat of loss to follow-up, the proposed design will use a three-pronged strategy. First, the project will offer food and transportation vouchers for those cohort members who attend the scheduled evaluations. Second, eligible participants will be informed ahead of time that the results from a comprehensive battery of clinical laboratory exams and cognitive and functional test results will be available free of charge to cohort participants at baseline and follow-up measurement times. Third, a cloud-based data collection platform will keep participants engaged by providing them with regular feedback on data collection, intervention activities, and study results and sending health educational messages through push notifications, phone calls, or text messages twice a month.

It is also possible that measuring factors in the IC domains, such as balance and gait in the mobility domain, may incur measurement errors. To address the misclassification of these exposure factors, our proposed design will use measurement and scales that are acceptable, standardized, and tested for validity (Beyene et al., 2024). Finally, we aim to minimize confounding in the analyses of measures of associations between IC and multiple factors by collecting and incorporating the most relevant contextual and risk factors for both IC and health outcomes in the statistical models.

## Data Availability

This article describes a study protocol for a future cohort study. No data have been collected or analyzed. When the study is completed, data availability will be determined according to ethical approval and participant consent agreements.
